# Systematic evaluation for effects of urine pH on calcium oxalate crystallization, crystal-cell adhesion and internalization into renal tubular cells

**DOI:** 10.1038/s41598-017-01953-4

**Published:** 2017-05-11

**Authors:** Juthatip Manissorn, Kedsarin Fong-ngern, Paleerath Peerapen, Visith Thongboonkerd

**Affiliations:** Medical Proteomics Unit, Office for Research and Development, Faculty of Medicine Siriraj Hospital; and Center for Research in Complex Systems Science, Mahidol University, Bangkok, Thailand

## Abstract

Urine pH has been thought to be an important factor that can modulate kidney stone formation. Nevertheless, there was no systematic evaluation of such pH effect. Our present study thus addressed effects of differential urine pH (4.0–8.0) on calcium oxalate (CaOx) crystallization, crystal-cell adhesion, crystal internalization into renal tubular cells, and binding of apical membrane proteins to the crystals. Microscopic examination revealed that CaOx monohydrate (COM), the pathogenic form, was crystallized with greatest size, number and total mass at pH 4.0 and least crystallized at pH 8.0, whereas COD was crystallized with the *vice versa* order. Fourier-transform infrared (FT-IR) spectroscopy confirmed such morphological study. Crystal-cell adhesion assay showed the greatest degree of crystal-cell adhesion at the most acidic pH and least at the most basic pH. Crystal internalization assay using fluorescein isothiocyanate (FITC)-labelled crystals and flow cytometry demonstrated that crystal internalization into renal tubular cells was maximal at the neutral pH (7.0). Finally, there were no significant differences in binding capacity of the crystals to apical membrane proteins at different pH. We concluded that the acidic urine pH may promote CaOx kidney stone formation, whereas the basic urine pH (i.e. by alkalinization) may help to prevent CaOx kidney stone disease.

## Introduction

Urine compositions can be used to evaluate the stone risk and to monitor therapeutic response in patients with nephrolithiasis/urolithiasis^1, 2^. The normal urine is slightly acidic with pH of approximately 6.0 although it can range from 4.5 to 8.0^3, 4^. The urine pH has been observed to be associated with many diseases, i.e. urothelial carcinoma, metabolic disorders, and kidney stone disease^5–7^. In nephrolithiasis/urolithiasis, urine pH has been thought to modulate kidney stone formation at various steps, including crystallization, growth, aggregation and retention^8–10^. In addition, pH is an important factor that can enhance the generation of solid phase and affects solubility of kidney stones^11, 12^. Moreover, several stone types, including calcium oxalate (CaOx), calcium phosphate, uric acid, etc., have been reported to be modifiable by urine pH^1, 8, 12^. There are some previous studies reporting evidence of the pH-dependent formation renal calculi^13, 14^. The basic urine pH favors formation of phosphate-containing stones, whereas the acidic urine pH is associated with uric acid and cystine stones^13, 14^.

CaOx is the most common type of stones removed from the stone formers^15, 16^. The most common hydrate form of CaOx crystals found inside the stone matrix is CaOx monohydrate (COM or whewellite), whereas CaOx dihydrate (COD or weddellite) is the second^17^. Between these two forms, COM is more pathogenic, whereas COD is more physiologic, as the latter can be found in the urine of healthy individuals when it is concentrated but the former or pathogenic form is usually found in the urine of the stone formers^18–20^. Several compositions in the urine can either promote or inhibit CaOx stone formation, such as calcium, oxalate, citrate, proteins, macromolecules, glycosaminoglycan, and pH^2, 7, 21–23^.

Although urine pH has been well recognized as one of the modulators for kidney stone formation, nevertheless, there was no systematic evaluation of such pH effects. In this present study, we thus examined effects of differential urine pH (from 4.0 to 8.0) on CaOx crystallization, crystal-cell adhesion, crystal internalization into renal tubular cells, and binding of apical membrane proteins to the crystals.

## Results

### Effect of urine pH on CaOx crystallization

**Figure 1:**
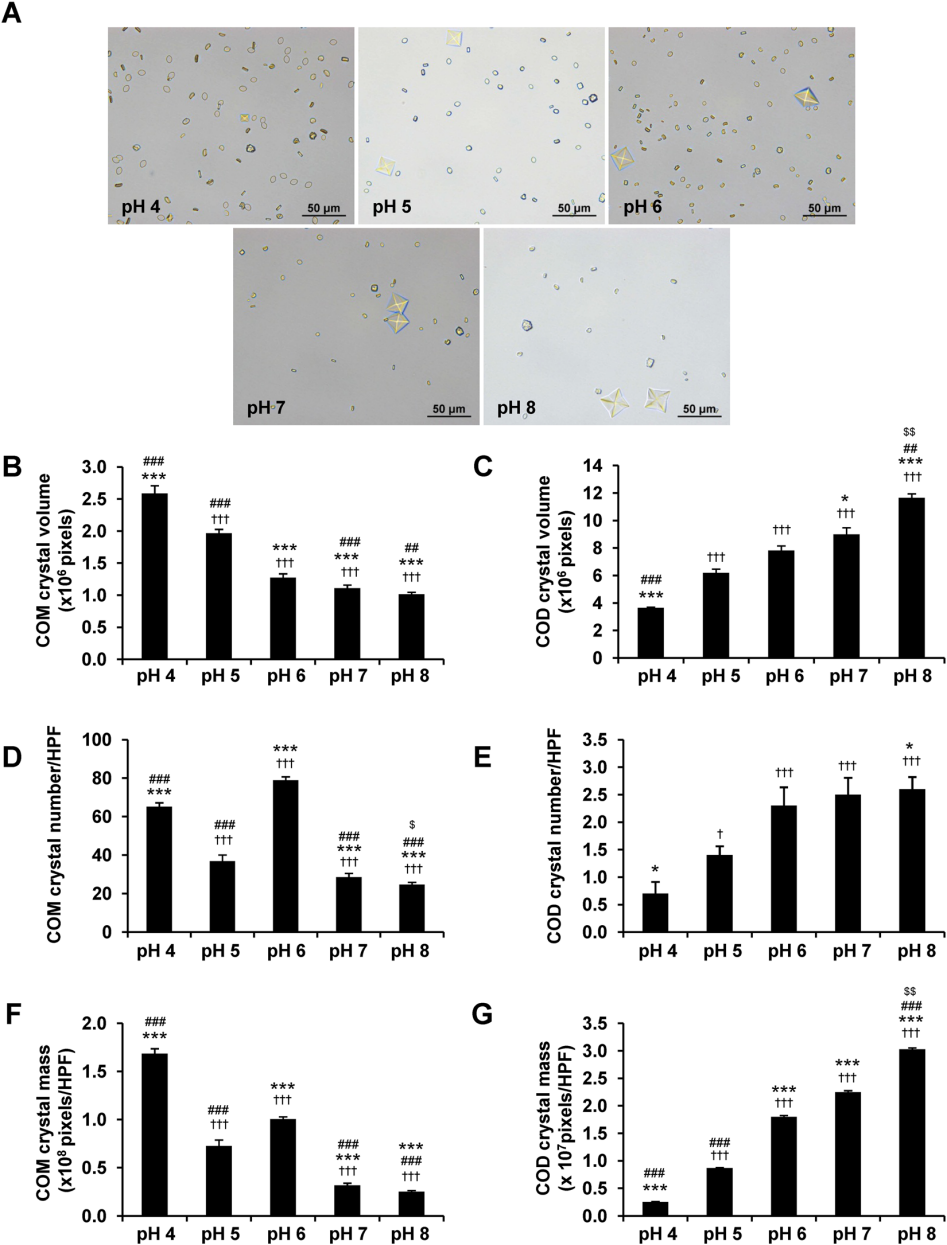
Effect of urine pH on CaOx crystallization. CaOx was crystallized in AU with varying pH (from 4.0 to 8.0). (**A**) Morphology of the resulting CaOx crystals (original magnification power was 400×). (**B**) COM crystal size (volume) measured from at least 100 crystals/each condition in each replicate. (**C**) COD crystal size measured from at least 20 crystals/each condition in each replicate. (**D**,**E**) Number of COM and COD crystals, respectively, counted from at least 10 HPF/each condition in each replicate. (**F**,**G**) COM and COD crystal masses (calculated from crystal size × crystal number). The data are reported as mean ± SEM of the data obtained from 3 independent experiments. ^†^ *p* < 0.05 vs. pH 4; ^†††^ *p* < 0.001 vs. pH 4; *p < 0.05 vs. pH 5; ***p < 0.001 vs. pH 5; ^##^ *p* < 0.01 vs. pH 6; ^###^ *p* < 0.001 vs. pH 6; ^$^ *p* < 0.05 vs. pH 7; ^$$^ *p* < 0.01 vs. pH 7.

Several previous studies have shown that CaOx crystals can easily form inside distal renal tubules and urine pH is a crucial factor affecting such crystallization^24–27^. The difference of urine pH could generate various CaOx crystal types^14^. In this experiment, we evaluated the effect of differential pH (from 4.0 to 8.0) on CaOx crystallization in artificial urine (AU). Morphological examination showed that COM (with monoclinic prismatic shape) was crystallized most effectively at the most acidic pH (at 4.0) and least at the most basic pH (at 8.0) (Fig. 1A). *Vice versa*, COD (with bipyramidal shape) was crystallized most effectively at the most basic pH (at 8.0) and least at the most acidic pH (at 4.0) (Fig. 1A). Quantitative data of crystal size (volume), number and mass were consistent with the morphological appearance (Fig. 1B–G).

**Figure 2:**
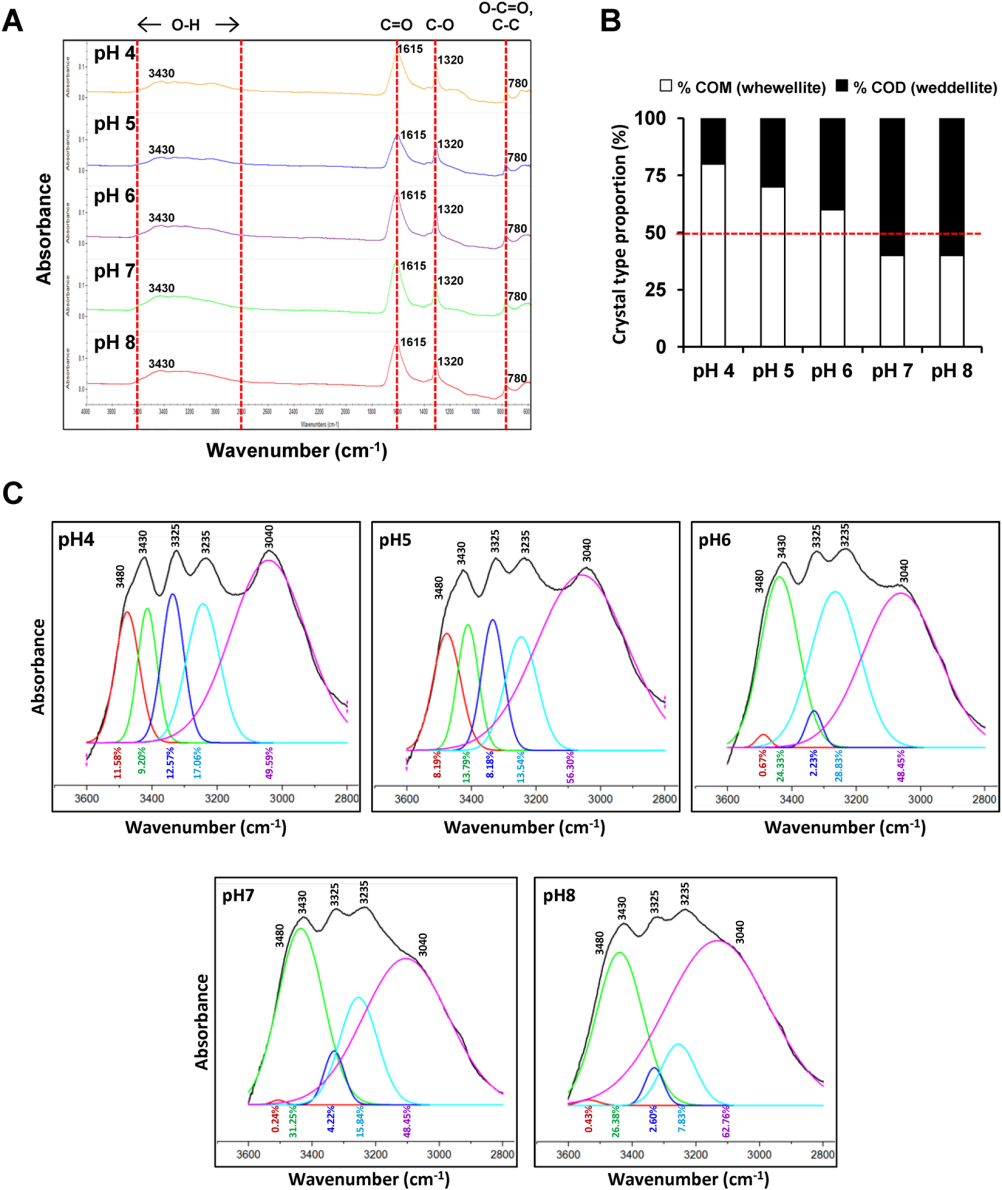
FT-IR spectra of CaOx crystallized in AU with various pH. (**A**) FT-IR spectra demonstrated the consistent spectra of CaOx. (**B**) Proportion of CaOx monohydrate (COM or whewellite) and dihydrate (COD or weddellite) slightly varied among different pH conditions. (**C**) Curve fitting analysis to differentiate pattern of “O-H” stretching between wavenumber of 3600–2800 cm^−1^ of FT-IR spectra.

In addition, the effect of urine pH on CaOx crystallization was also analyzed and quantitated by Fourier-transform infrared (FT-IR) spectroscopy. There were four common regions of FT-IR spectra (at 3600–2800, 1615, 1315 and 780 cm^−1^) of the resulting CaOx crystallized at pH of 4.0–8.0 (Fig. 2A). Comparing these spectra with the FT-IR library revealed significant match of our data with the reference spectra of COM (whewellite) combined with COD (weddellite) with various proportions in differential pH (Fig. 2B). Semi-quantitative analysis of these data revealed combined COM and COD in all conditions with the greatest percentage of COM at the most acidic pH (4.0) and COD at the most basic pH (8.0) (Fig. 2B). *Vice versa*, the least proportion of COM was yielded at the most basic pH (8.0), whereas that of COD was obtained at the most acidic pH (4.0) (Fig. 2B). Furthermore, all the FT-IR spectra were also subjected to peak separation accomplished by curve fitting analysis. We focused our attention on the FT-IR spectra within the region of 3600–2800 cm^−1^ wavenumber because this is the best representative region for COM and COD differentiation^28, 29^. A total of five peaks were found within this region of the FT-IR spectra derived from the CaOx crystal mixture (Fig. 2C). Curve fitting analysis showed COM as a predominant crystal type at the acidic urine pH (4.0 and 5.0), whereas COD was predominant at other pH conditions (6.0–8.0) (Fig. 2C). These data were consistent with the semi-quantitative data obtained from matching with the reference FT-IR spectra of COM and COD as described above (Fig. 2B).

**Figure 3:**
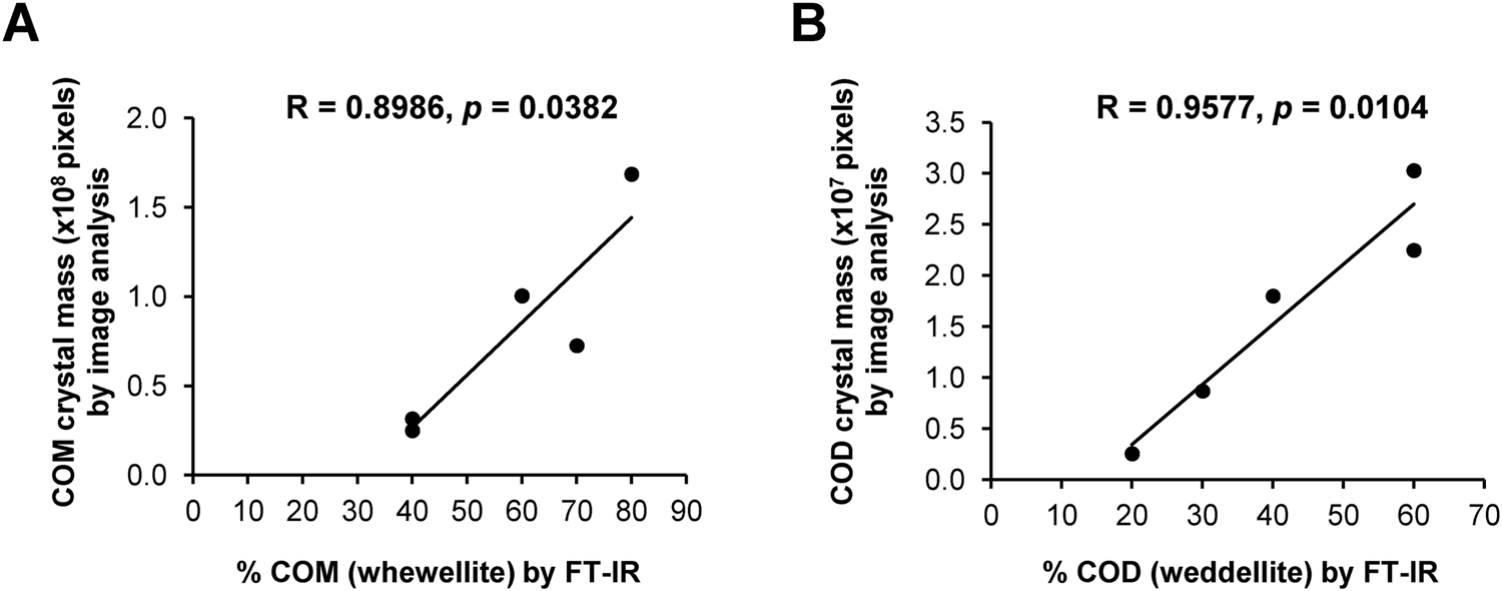
Correlation between CaOx crystal mass derived from morphological analysis and percentage of the crystal contents analyzed by FT-IR spectroscopy. Pearson’s correlation analysis revealed strongly positive correlation between CaOx crystal mass by image analysis and percentage of the crystal contents analyzed by FT-IR spectroscopy for both COM (**A**) and COD (**B**).

Moreover, we also evaluated a correlation between the crystal mass data of COM and COD derived from morphological examination (Fig. 1F and G) and their proportions in the crystal mixture analyzed by FT-IR spectroscopy (Fig. 2B). Pearson’s correlation analysis revealed strongly positive correlation between CaOx crystal mass obtained by image analysis and the percentage of crystal contents analyzed by FT-IR spectroscopy with coefficient of correlation (R) of 0.8986 (*p* < 0.05) and 0.9577 (*p* < 0.05) for COM and COD, respectively (Fig. 3). These findings indicated that the data obtained from morphological examination and FT-IR analysis were highly consistent.

### Effect of pH on renal tubular cell proliferation and death

**Figure 4:**
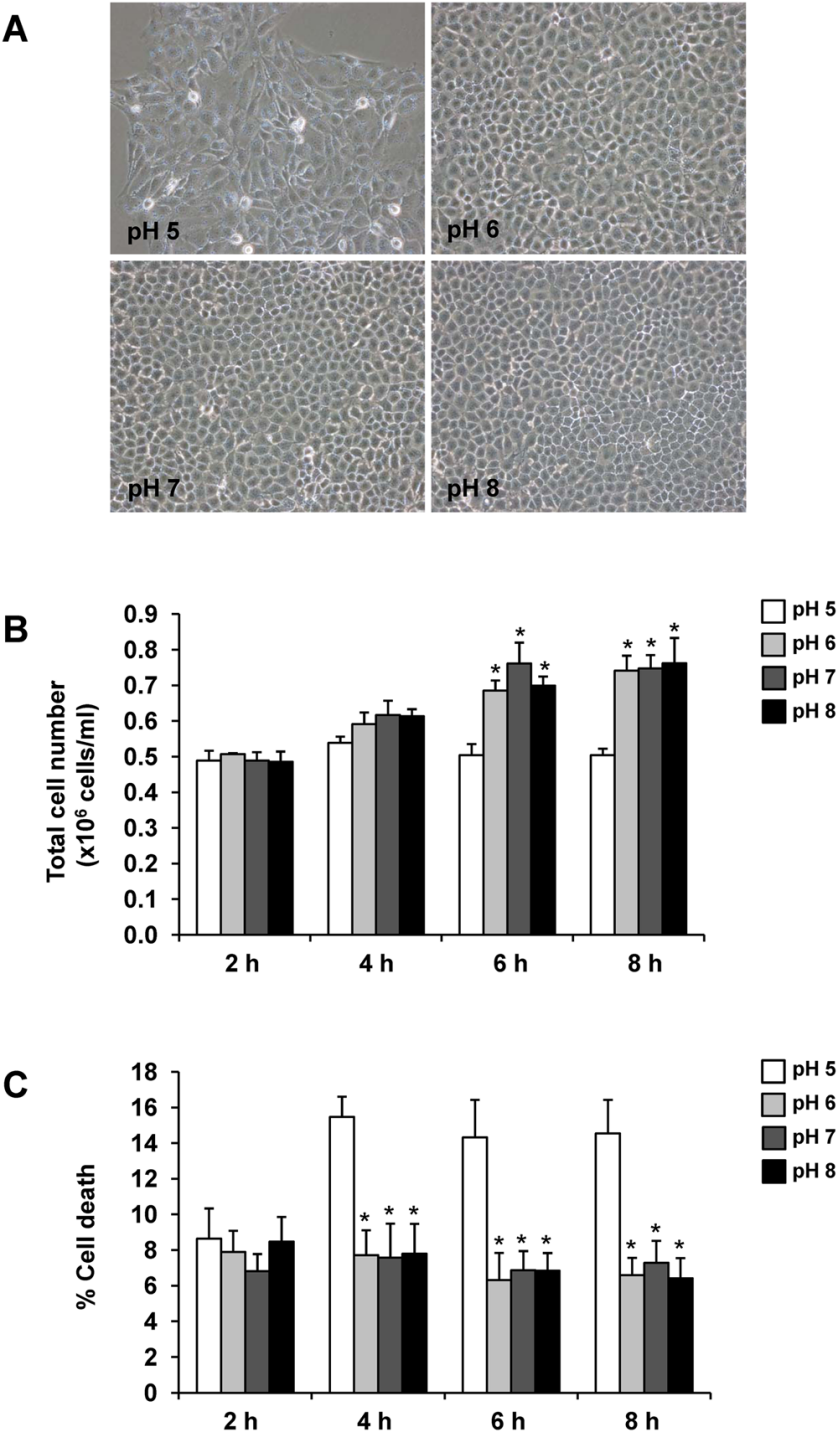
Effect of pH on renal tubular cell proliferation and death. MDCK cells were cultivated and maintained in medium with different pH (from 5.0 to 8.0) for up to 8 h. (**A**) Cell morphology observed under a phase contrast microscope at 8 h with original magnification of 200×. (**B**) Total cell number representing cell proliferation. (**C**) Cell death assay using Trypan blue staining. The data are reported as mean ± SEM of the data obtained from 3 independent experiments. *p < 0.05 vs. pH 5.

Madin-Darby Canine Kidney (MDCK) cell line originated from distal renal tubular segment^30, 31^ was used to evaluate effect of pH on renal tubular cells. Figure 4A showed the morphology of MDCK cells in various pH conditions (from 5.0 to 8.0) for up to 8 h. The results showed that MDCK cells at 8 h looked unhealthy at pH of 5.0 but looked normal at pH of 6.0–8.0. Note that the cells were also cultivated at pH of 4.0. However, they were too toxic and could not tolerate with such highly acidic pH. As such, the pH of 4.0 was excluded from all subsequent experiments dealing with renal tubular cells. For cell proliferation, the cell number significantly increased at 6-h and 8-h for pH of 6.0, 7.0 and 8.0. However, the cell number was not increased at all with pH 5.0, indicating defective cell proliferation at the most acidic pH (Fig. 4B). Cell death was not altered at pH of 6.0, 7.0 and 8.0, but was significantly increased at pH of 5.0 starting from 4-h after incubation through the end of the study (Fig. 4C).

### Effect of pH on COM crystal adhesion on renal tubular cells

**Figure 5:**
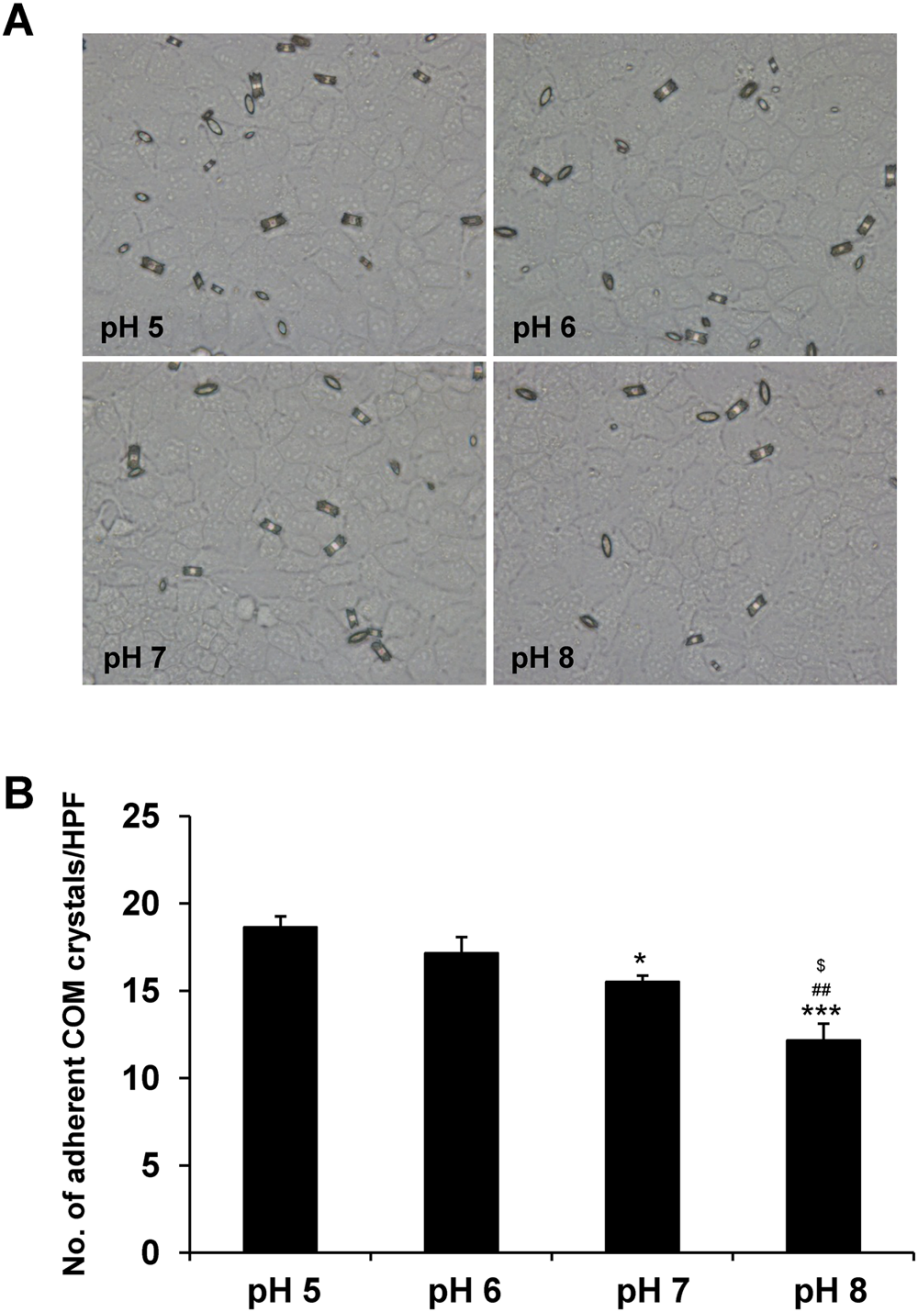
Effect of pH on COM crystal adhesion on renal tubular cells. Crystal-cell adhesion assay was performed using plain COM crystals under varying pH (from 5.0 to 8.0). (**A**) Microscopic examination of the crystals remained and adhered on the MDCK cell monolayer after washing in each condition (original magnification power was 400×). (**B**) Quantitative data of the adherent COM crystals from at least 15 randomized high-power fields (HPF) per culture well in each replicate. The data are reported as mean ± SEM of the data obtained from 3 independent experiments. *p < 0.05 vs. pH 5; ***p < 0.001 vs. pH 5; ^##^ *p* < 0.01 vs. pH 6; ^$^ *p* < 0.05 vs. pH 7.

Adhesion of the causative crystals on renal tubular epithelial cells has been thought to be a necessary event for kidney stone formation. Urine pH has been suggested to affect crystal-cell adhesion^32^. We thus evaluated the effect of pH on crystal-cell interaction by using crystal-cell adhesion assay. The data showed that differential pH dramatically affected the adhesive capability between the COM crystals and MDCK renal tubular cells in a pH-dependent manner with the most potent adhesive capability at the highly acidic pH (5.0) and least at the most basic pH (8.0) (Fig. 5).

### Effect of pH on internalization of COM crystals into renal tubular cells

**Figure 6:**
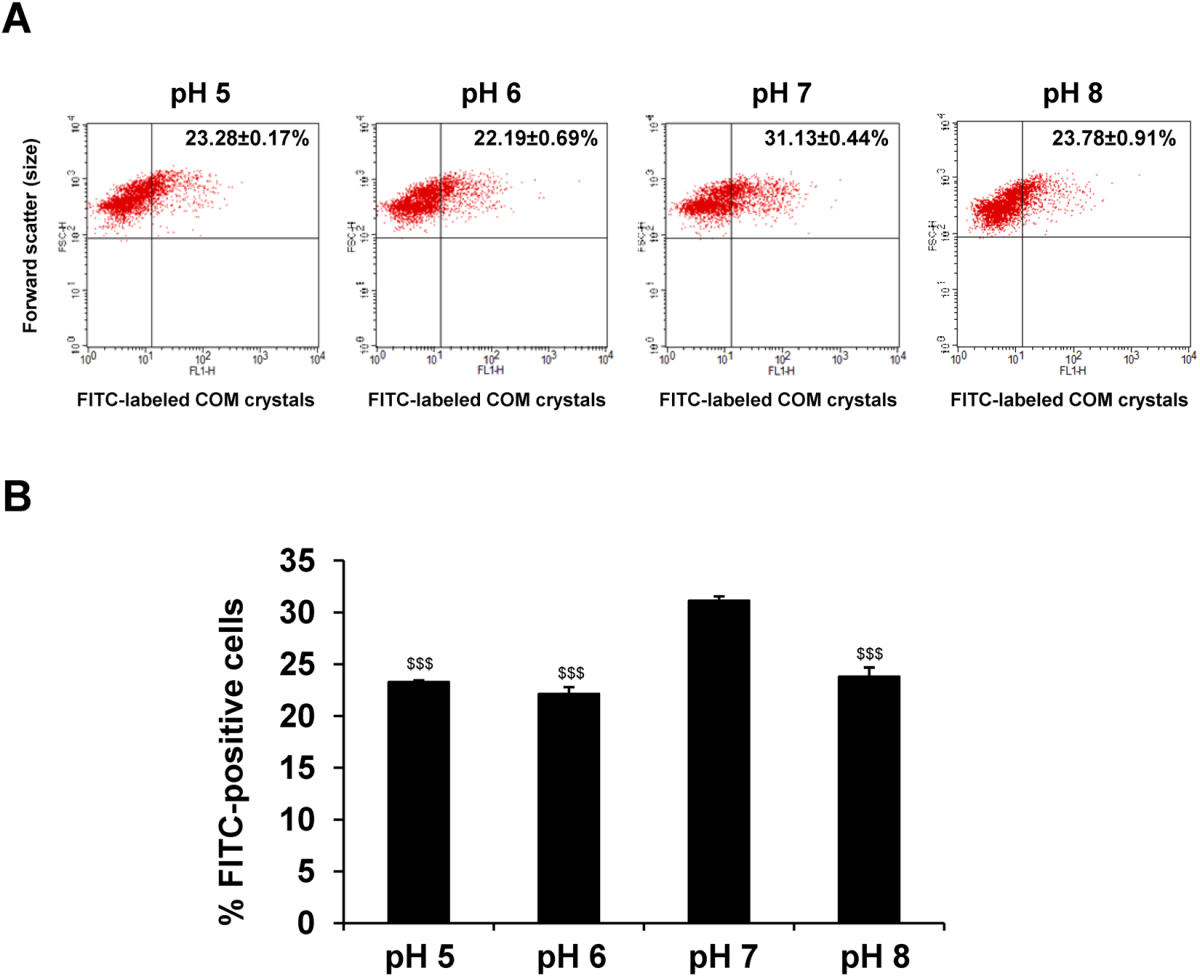
Effect of pH on internalization of COM crystals into renal tubular cells. Crystal internalization assay was performed using FITC-labelled COM crystals under varying pH (from 5.0 to 8.0). (**A**) Representative flow cytometric data of the internalized FITC-labeled COM crystals that were endocytosed by MDCK cells under varying pH conditions. (**B**) Quantitative data of FITC-positive cells. The data are reported as mean ± SEM of the data obtained from 3 independent experiments. ^$$$^ *p* < 0.001 vs. pH 7.

After COM crystals adhered on renal tubular cells, the crystals were subsequently endocytosed by the cells^33, 34^ and underwent degradation by cellular endolysosomal mechanisms^35^. We also hypothesized that urine pH might also affect crystal endocytosis into the cells. We thus evaluated the effect of urine pH on crystal internalization using FITC-labelled COM crystals and flow cytometry. The results showed that percentage of the cells with endocytosed crystals was obviously increased only at pH of 7.0, whereas other pH levels had comparable crystal internalization (Fig. 6). Surprisingly, internalization was not pH-dependent but was maximal at the neutral pH. This might reflect the normal cell physiology that favors internalization of the adhered crystals for subsequent degradation as a defense mechanism of the cells to control homeostasis mostly at the neutral pH.

### Effect of pH on COM-binding capacity of apical membrane proteins

**Figure 7:**
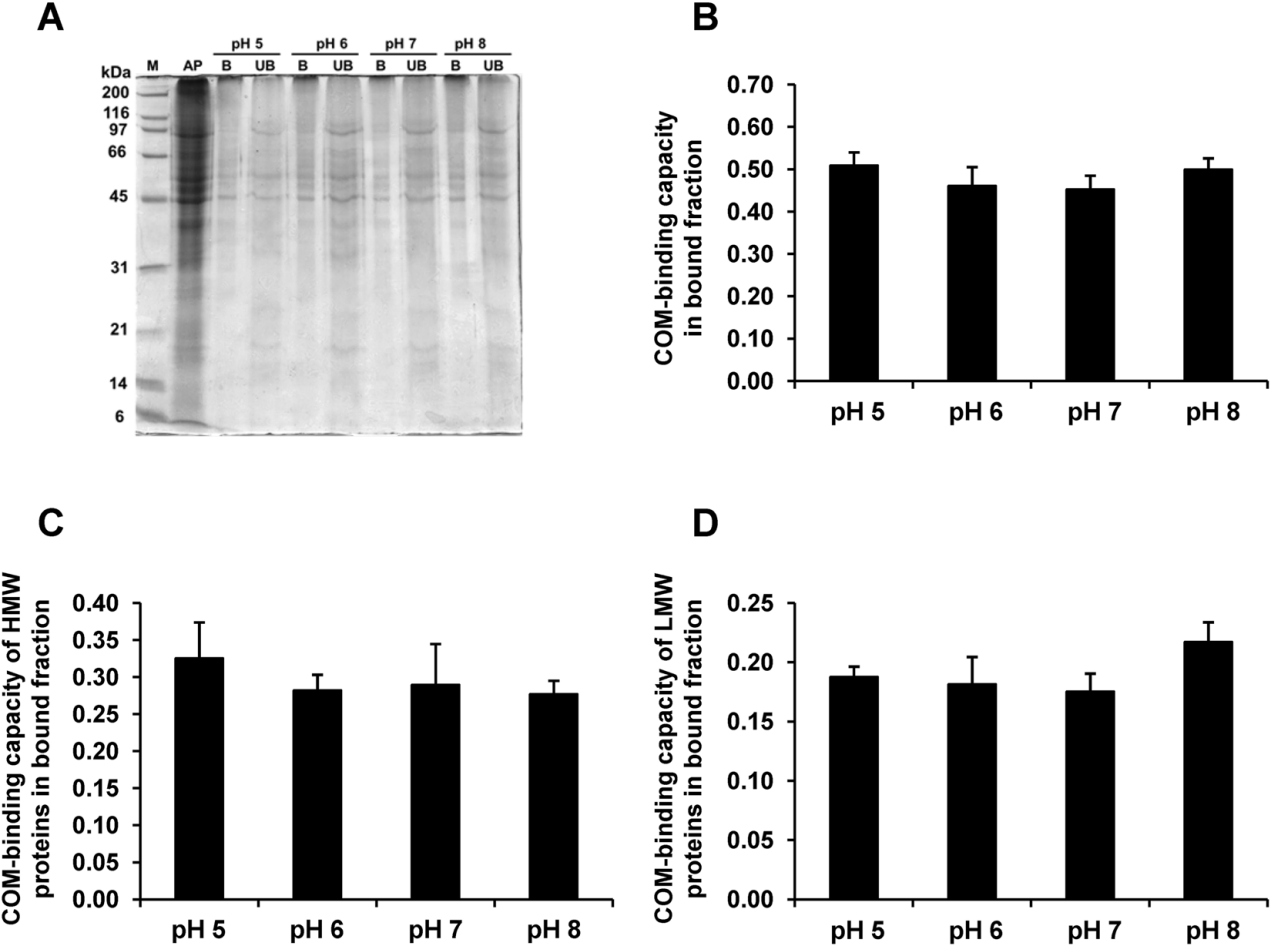
Effect of pH on COM-binding capacity of apical membrane proteins. Proteins isolated from apical membranes of polarized MDCK cells were incubated with plain COM crystals under varying pH conditions. (**A**) SDS-PAGE band patterns of apical membrane (AP), bound (B) and unbound (UB) fractions stained with Coomassie Brilliant Blue G-250. (**B**) COM-binding capacity of proteins in the bound fraction. (**C**) COM-binding capacity of HMW proteins (M_r_ ≥ 45 kDa) in bound fraction. (**D**) COM-binding capacity of LMW proteins (M_r_ < 45 kDa) in bound fraction. The data are reported as mean ± SEM of the data obtained from 3 independent experiments.

Apical membranes were isolated, solubilized and incubated with COM crystals at differential pH (from 5.0 to 8.0). Proteins from the bound and unbound fractions were the resolved with 12% SDS-PAGE and visualized by Coomassie Brilliant Blue G-250 staining (Fig. 7A). The COM-binding capacities of all proteins, HMW proteins and LMW proteins in the bound fraction were then calculated using the formulas described in “Materials and Methods”. The quantitative data revealed no significant differences of the COM-binding capacities of all proteins, HMW proteins and LMW proteins in the bound fraction under differential pH conditions (Fig. 7B,C). These data indicated that urine pH did not affect compositions of the proteins bound to the crystals. This strengthened the hypothesis mentioned above that the acidic pH caused cellular toxicity that finally led to membrane lipid asymmetry and finally enhancement of crystal-cell adhesion^36, 37^.

## Discussion

Urine pH is one the major factors affecting kidney stone formation and different pH levels are associated with specific types of the renal calculi^13, 14^. We first evaluated the effect of differential pH (from 4.0 to 8.0) on CaOx crystallization in AU by performing morphological examination using a three-dimensional (3D) mode of Image Master 2D Platinum software to obtain the crystal volume data. Crystal mass was calculated by multiplying an average crystal size (volume) by crystal number. COM was crystallized most effectively at the most acidic pH (4.0), whereas COD was crystallized most effectively at the most basic pH (at 8.0) (Fig. 1).

FT-IR spectroscopy is one of powerful tools for investigation of chemical compositions in a complex mixture. Each FT-IR spectrum represented the stretching vibration of each functional group. Four common regions of the FT-IR spectra were derived from a mixture of CaOx crystals as shown in Fig. 2A. This FT-IR spectral pattern was in concordance with several lines of evidence reporting the signal stretching vibration of O-H group at 3600–2800 cm^−1^, C=O group at 1615 cm^−1^, C-O group at 1315 cm^−1^, and O-C=O/C-C group at 780 cm^−1^ in the CaOx molecule^38, 39^. Fortunately, COM and COD crystals could be discriminated from each other by curve fitting analysis using FT-IR spectra between 3600–2800 cm^−1^ wavenumber^28, 29^. There were five peaks derived from symmetric and asymmetric O-H stretching within this region of the wavenumber for CaOx crystals generated under various pH (Fig. 2C). Among these, a broad spectrum at 3040 cm^−1^, which was common for both COM and COD, was found in all pH conditions, whereas other four peaks could be used to differentiate COM from COD^28, 29^. In COM, the peaks at 3480, 3430, 3325 and 3235 cm^−1^ had comparable amplitude and configuration, and more importantly were well-defined and could be easily distinguished from the others (Fig. 2C). In contrast, these four peaks were much broader and had a wide range of amplitudes in COD. As a result, the COD peaks at 3480, 3430, 3325 and 3235 cm^−1^ were less defined and some of them were overlapped with the others (Fig. 2C). From curve fitting analysis of the O-H stretching region, the pattern consisting of five well-defined peaks representing COM was found in more acidic pH (4.0 and 5.0) and the broader pattern representing COD was found in other (more basic) pH (6.0–8.0). These FT-IR findings confirmed that COM favors crystallization at a strongly acidic pH, whereas COD is predominantly found in the more basic pH conditions.

Comparing to COD, COM is more pathogenic for kidney stone formation by its higher adhesive capability^40^ and its higher proportion found in stone matrices isolated from >111,000 stone formers^17^. The higher percentage of COD and less proportion of COM found at the most basic pH indicated that alkalization of the urine may decrease stability and increase solubility of COM crystals. Moreover, the alkalized urine may also facilitate transformation of COM to COD^40, 41^.

It should be noted that we generated CaOx crystals in the protein-free AU instead of the natural urine because we would like to eliminate confounding factors from a wide variety of urinary proteins that could easily interfere with the data interpretation^42^. Additionally, changes in the urine pH has been reported to directly alter various properties of urinary proteins, including their function, structure or conformation^43^. These suggested us to use protein-free AU in crystallization assay to eliminate the unavoidable interference from urinary proteins. As a result, our present study could focus only on the effects of urine pH on various steps of CaOx kidney stone formation.

MDCK cells at acidic pH (5.0 and 6.0) revealed the high affinity for COM crystal binding as compared to those exposed to neutral and basic pH (7.0 and 8.0) (Fig. 5). Tissue and cellular injury is one of the potential factors aggravating kidney stone formation^36, 37^. The injury can induce membrane lipid asymmetry and loss of cell polarity, as well as crystal-cell adhesion^36, 37^. Our data showed that the cells underwent cytotoxicity at the highly acidic pH (Fig. 4), at which crystal-cell adhesion was most potent (Fig. 5). These coordinated findings implicated that cellular injury might be a potential mechanism underlying the highly potent crystal-cell adhesive capability at the acidic pH. The enhancement of COM crystal binding affinity to the injured renal tubular epithelial cells might be the result of the asymmetry or unveiling of the membrane lipids, especially phosphatidylserine, a strong COM crystal-binding molecule, to their outer surface^44^.

In this study, COM crystal internalization was not pH-dependent. The highest degree of the crystal internalization was found at the neutral pH (7.0) (Fig. 6). This data implicated that the cell health status and homeostasis is an important factor affecting the ability of renal tubular epithelial cells to internalize COM crystals. Our data was consistent with the result from a previous study reporting the higher capacity of COM crystal internalization in healthy renal cells, as compared to the damaged cells^45^. Crystal internalization has been thought to be an active transport mechanism involving actin cytoskeleton-mediated macropinocytosis^34^. Therefore, this inferior ability of the injured renal cells and those exposed to non-physiologic pH (<7.0 and >7.0) might be due to defective assembly or disorganization of actin cytoskeleton inside the cells, leading to their defect in crystal internalization through macropinocytosis.

In summary, we have demonstrated by morphological examination and FT-IR spectroscopy that the acidic urine pH favors crystallization of COM, which is more pathogenic, whereas the basic urine pH favors crystallization of COD, which is more physiologic. Additionally the acidic urine pH is also associated with cellular injury and enhanced COM crystal-cell adhesion that may lead to crystal retention. Finally, internalization of COM crystals into the cells is most effective at the neutral pH. From these findings, alkalinization of the urine may help to prevent COM kidney stone formation and recurrence.

## Materials and Methods

### Preparation of plain COM crystals and fluorescence-labelled COM crystals

COM crystals were prepared according to protocols established previously^46, 47^. Briefly, 10 mM CaCl_2_·2H_2_O and 10 mM Na_2_C_2_O_4_ were mixed to make final concentrations of 5 mM and 0.5 mM, respectively, in Tris buffer containing 90 mM NaCl (pH 7.4). The mixture was incubated at room temperature (RT) (set at 25 °C) overnight and then centrifuged at 2,000 *g* for 5 min. COM crystal pellets were collected, resuspended in methanol and then centrifuged at 2,000 *g* for 5 min. Methanol was removed and COM crystals were air-dried. The fluorescence-labelled COM crystals were generated as previously described using 0.5 µg/mL fluorescein isothiocyanate (FITC) (Thermo Scientific; Waltham, MA)^35, 48, 49^. Both plain and FITC-labelled COM crystals were decontaminated by UV light radiation for 30 min before intervention with the cells.

### Preparation of artificial urine (AU)

Artificial urine was prepared according to protocols established recently^50^. The plain AU contained 200 mM urea, 54 mM NaCl, 30 mM KCl, 15 mM NH_4_Cl, 9 mM Na_2_SO_4_, 5 mM CaCl_2_, 5 mM Na_3_C_6_H_5_O_7_·2H_2_O, 4 mM creatinine, and 2 mM MgSO_4_·7H_2_O with pH = 6.2 and osmolality = 446 mOsm/kg^50^. The urine pH was then adjusted to 4.0–8.0 using NaOH and HCl.

### CaOx crystallization assay

CaOx was freshly crystallized (with 10 mM CaCl_2_·2H_2_O and 10 mM Na_2_C_2_O_4_ to make final concentrations of 5 mM and 0.5 mM, respectively) in AU with urine pH of 4.0–8.0 in 24-well plate. The solution was gently mixed and incubated at RT for 1 h. Crystal morphology was observed and their images were taken under a bright-field microscope connected with a digital camera (Nikon Eclipse Ti-S, Nikon; Tokyo, Japan). Crystal size was analyzed by Image Master 2D Platinum software (GE Healthcare; Uppsala, Sweden) using a three-dimensional (3D) mode to obtain the raw data of CaOx crystal volume reported in pixel unit from at least 100 individual COM crystals and at least 20 individual COD crystals for each condition. Number of CaOx crystals was counted from at least 10 high-power fields (HPFs) and total crystal mass was calculated from the data obtained from at least 10 HPFs by using the following formula:

1 $$\begin{array}{rcl}Crystal\,mass\,(pixel/HPF) & = & Average\,crystal\,size\,(volume)\,in\,each\,HPF\,(pixel)\\  &  & \times \,Crystal\,number\,in\,each\,HPF\,(/HPF)\end{array}$$

### Fourier-transform infrared (FT-IR) spectroscopy

In addition to morphological examination, crystal type was also confirmed and quantitated by Fourier-transform infrared (FT-IR) spectroscopy. Briefly, the precisely equal amount (1.5 mg) of CaOx crystals generated in AU with various pH (as detailed above) were analyzed by FT-IR spectroscopy equipped with an attenuated total reflectance (ATR) accessory and OMNIC software version 8.3 (Nicolet 6700, Thermo Scientific Inc.; Waltham, MA). The crystal spectra were acquired from 4000–600 cm^−1^, in which 64 scans were averaged with a 4 cm^−1^ resolution for each spectrum. All the resulting spectra were then compared with the reference FT-IR spectra for kidney stone, namely the “Kidney Stone Library – Basic”, consisting of more than 800 spectra of pure and mixed (with various proportions) COM and COD contents. The proportion of COM and COD contents of the most identical spectra that provided the highest matched score was then reported as a semi-quantitative data. Curve fitting analysis was further investigated by using Origin software (OriginLab; Northampton, MA) using the Gaussian function.

### Renal tubular cell cultivation

Madin-Darby Canine Kidney (MDCK) cell line originated from distal renal tubular segment^30, 31^ was used in this study. MDCK cells were grown in Eagle’s minimum essential medium (MEM) (Gibco, Invitrogen Corporation; Grand Island, NY) supplemented with 10% fetal bovine serum (FBS), 1.2% penicillinG/streptomycin and 2 mM L-glutamine and maintained in a humidified incubator with 5% CO_2_ at 37 °C. Note that the pH of cell culture medium was adjusted to 5.0, 6.0, 7.0 or 8.0 using NaOH and HCl. Cell morphology was observed under a phase contrast microscope (Olympus CKX41; Tokyo, Japan).

### Cell proliferation and death assay

MDCK cells were cultivated in various pH as mentioned above. After cultivation for 2, 4, 6 and 8 h, the cells were detached using 0.1% trypsin in 2.5 mM EDTA. Trypsin activity was terminated by using FBS-containing MEM. The cell suspension was then mixed with 0.4% trypan blue solution (Gibco, Invitrogen Corporation) and both viable and dead cells were counted using a hemacytometer.

### Crystal-cell adhesion assay

MDCK cells were inoculated in FBS-containing MEM for 48 h. The culture medium was then removed and MDCK cells were washed with PBS twice. Crystal-cell adhesion was initiated by adding 100 µg/mL plain COM crystals (in FBS-containing MEM with pH adjusted to 5.0, 6.0, 7.0 or 8.0) into each culture well. The cells were further incubated in a humidified incubator at 37 °C with 5% CO_2_ for 1 h. Thereafter, the cells were vigorously washed with PBS five times to remove unbound COM crystals. Finally, the adhered crystals remained on the cell monolayer were counted from at least 15 randomized high-power fields (HPF) per culture well under a bright-field microscope (Nikon Eclipse Ti-S, Nikon).

### Crystal internalization assay

MDCK cells were grown in 35-cm^2^ tissue culture dishes for quantitative analysis of the internalized crystals by flow cytometry using FITC-labelled COM crystals. After 48-h maintenance, the plain culture medium was replaced with MEM containing FITC-labelled COM crystals (500 µg of crystals/mL of culture medium) in various pH (from 5.0 to 8.0). After 1-h incubation, the medium was removed and the cells were washed with PBS. The cells were then incubated with 5 mM EDTA/PBS for 5 min to eliminate non-internalized crystals and further incubated with trypsin-EDTA solution to detach the cells. The cells with internalized FITC-labelled COM crystals were quantified by using a flow cytometer (FACScan, Becton Dickinson Immunocytometry System; San Jose, CA).

### Crystal-protein binding assay

MDCK cells at a density of approximately 5.0–7.5 × 10^4^ cells/ml were seeded and grown on prewetted collagen-coated permeable polycarbonate membrane in Transwells^TM^ (0.4 µm pore size; Corstar; Cambridge, MA) for four days to develop polarization (note that the culture medium was refreshed every other day). The culture medium was then removed and apical membranes of the polarized MDCK cells were isolated by a peeling method established recently^51, 52^. Briefly, the polarized MDCK cells were rinsed twice with ice-cold membrane-preserving buffer (1 mM MgCl_2_ and 0.1 mM CaCl_2_ in PBS). Thereafter, Whatman filter paper (0.18-mm-thick, Whatman International Ltd.; Maidstone, UK) pre-wetted with deionized water was placed onto the polarized cell monolayer. After a 5-min incubation period, the filter paper was peeled out and the apical membranes retained at the filter paper surface were harvested by rehydration in deionized water and gentle scrapping. The isolated apical membranes were lyophilized and solubilized in Laemmli’s buffer.

The protein solution was then dialyzed against deionized water and then lyophilized. Finally, apical membrane proteins were resuspended in 1 mL AU with varying pH (from 5.0 to 8.0) and incubated with COM crystals (5 mg crystals per mL AU) at 4 °C for 24 h using continuous rotator. The crystal-protein complexes were then collected by a centrifugation at 2,000 *g* for 5 min at 4 °C and the unbound proteins were collected. Thereafter, the crystal-protein complexes were washed three times with PBS and other three times with 4 mM EDTA in PBS. After the final wash with PBS, COM crystal-binding proteins were eluted by Laemmli’s buffer. Both unbound and bound fractions were resolved in 12% SDS-PAGE gel and the protein band patterns were visualized by Coomassie Brilliant Blue G-250 stain.

Protein band intensity was analyzed using ImageQuant TL software (GE Healthcare). COM-binding capacity was calculated by the following formulas:

2 $$\begin{array}{rcl}COM-binding\,capacity\,of\,bound\,fraction & = & Intensity\,of\,all\,bands\,in\,bound\,fraction\\  &  & /Intensity\,of\,all\,bands\,in\\  &  & \,both\,bound\,{\&}\,unbound\,fractions\end{array}$$

3 $$\begin{array}{rcl}\begin{array}{c}COM-binding\,capacity\,of\,HMW\\ proteins\,in\,bound\,fraction\end{array} & = & Intensity\,of\,protein\,bands\,with\,\\  &  & {M}_{r}\ge 45\,kDa\,in\,bound\,fraction\\  &  & \begin{array}{c}/Intensity\,of\,all\,bands\,in\,both\\ bound\,{\&}\,unbound\,fractions\end{array}\end{array}$$

4 $$\begin{array}{rcl}\begin{array}{c}COM-binding\,capacity\,of\,LMW\\ proteins\,in\,bound\,fraction\end{array} & = & Intensity\,of\,protein\,bands\,with\,\\  &  & {M}_{r} < 45\,kDa\,in\,bound\,fraction\\  &  & \begin{array}{c}/Intensity\,of\,all\,bands\,in\,both\\ bound\,{\&}\,unbound\,fractions\end{array}\end{array}$$

### Statistical analysis

The data are presented as mean ± SEM derived from three independent experiments. Comparisons between two groups of the samples were performed using unpaired Student’s *t*-test, whereas multiple comparisons of more than two groups of samples were performed using one-way analysis of variance (ANOVA) with Tukey’s post-hoc test. Pearson’s correlation test was performed to evaluate relationship between crystal mass of COM and COD derived from morphological examination and their proportions in the crystal mixture analyzed by FT-IR spectroscopy. *P*-values less than 0.05 were considered statistically significant.

## References

[1] Grases F, Costa-Bauza A, Prieto RM (2006). Renal lithiasis and nutrition. Nutr. J.

[2] Barbas C, Garcia A, Saavedra L, Muros M (2002). Urinary analysis of nephrolithiasis markers. J. Chromatogr. B Analyt. Technol. Biomed. Life Sci.

[3] Welch AA, Mulligan A, Bingham SA, Khaw KT (2008). Urine pH is an indicator of dietary acid-base load, fruit and vegetables and meat intakes: results from the European Prospective Investigation into Cancer and Nutrition (EPIC)-Norfolk population study. Br. J Nutr..

[4] Thongboonkerd V, Mungdee S, Chiangjong W (2009). Should urine pH be adjusted prior to gel-based proteome analysis?. J Proteome. Res..

[5] Ide H (2016). Urinary pH Levels are Strongly Associated with Bladder Recurrence After Nephroureterectomy in Upper Tract Urothelial Carcinoma Patients with a Smoking History. Ann. Surg. Oncol..

[6] Cho YH (2014). The association between a low urine pH and the components of metabolic syndrome in the Korean population: Findings based on the 2010 Korea National health and nutrition examination survey. J Res. Med Sci.

[7] Bihl G, Meyers A (2001). Recurrent renal stone disease-advances in pathogenesis and clinical management. Lancet.

[8] Tiselius HG (2011). A hypothesis of calcium stone formation: an interpretation of stone research during the past decades. Urol. Res.

[9] Han H, Segal AM, Seifter JL, Dwyer JT (2015). Nutritional Management of Kidney Stones (Nephrolithiasis). Clin. Nutr. Res..

[10] Ratkalkar VN, Kleinman JG (2011). Mechanisms of Stone Formation. Clin. Rev. Bone Miner. Metab.

[11] Worcester, E. M. & Coe, F. L. Nephrolithiasis. *Prim*. *Care* **35**, 369–91, doi:10.1016/j.pop.2008.01.005, vii (2008). 10.1016/j.pop.2008.01.005 PMC2518455 18486720

[12] McKay CP (2010). Renal stone disease. Pediatr. Rev..

[13] Grases F (2012). Urinary pH and renal lithiasis. Urol. Res..

[14] Wagner CA, Mohebbi N (2010). Urinary pH and stone formation. J Nephrol..

[15] Tsujihata M (2008). Mechanism of calcium oxalate renal stone formation and renal tubular cell injury. Int. J Urol..

[16] Ivanovski O, Drueke TB (2013). A new era in the treatment of calcium oxalate stones?. Kidney Int..

[17] Schubert G (2006). Stone analysis. Urol. Res..

[18] Saetun P, Semangoen T, Thongboonkerd V (2009). Characterizations of urinary sediments precipitated after freezing and their effects on urinary protein and chemical analyses. Am. J Physiol Renal Physiol.

[19] Thongboonkerd V (2008). Proteomics and kidney stone disease. Contrib. Nephrol..

[20] Vinaiphat A, Thongboonkerd V (2017). Prospects for proteomics in kidney stone disease. Expert Rev. Proteomics.

[21] Khan SR (2004). Role of renal epithelial cells in the initiation of calcium oxalate stones. Nephron Exp. Nephrol..

[22] Kumar V, Farell G, Lieske JC (2003). Whole urinary proteins coat calcium oxalate monohydrate crystals to greatly decrease their adhesion to renal cells. J. Urol..

[23] Kumar V, Lieske JC (2006). Protein regulation of intrarenal crystallization. Curr. Opin. Nephrol. Hypertens..

[24] Berg C, Tiselius HG (1986). The effect of pH on the risk of calcium oxalate crystallization in urine. Eur. Urol..

[25] Hojgaard I, Fornander AM, Nilsson MA, Tiselius HG (1998). The influence of hydroxyapatite seed on the crystallization induced by volume reduction of solutions with an ion composition corresponding to that in the distal tubule at different pH levels. Scand. J Urol. Nephrol..

[26] Hojgaard I, Fornander AM, Nilsson MA, Tiselius HG (1999). The effect of pH changes on the crystallization of calcium salts in solutions with an ion composition corresponding to that in the distal tubule. Urol. Res..

[27] Hojgaard I, Tiselius HG (1999). Crystallization in the nephron. Urol. Res..

[28] Frost RL (2004). Raman spectroscopy of natural oxalates. Anal. Chim. Acta..

[29] Conti C (2010). Stability and transformation mechanism of weddellite nanocrystals studied by X-ray diffraction and infrared spectroscopy. Phys. Chem. Chem. Phys..

[30] Rindler MJ, Chuman LM, Shaffer L, Saier MH (1979). Retention of differentiated properties in an established dog kidney epithelial cell line (MDCK). J Cell Biol..

[31] Saier MH (1981). Growth and differentiated properties of a kidney epithelial cell line (MDCK). Am. J Physiol.

[32] Wiessner JH, Hung LY, Mandel NS (2003). Crystal attachment to injured renal collecting duct cells: influence of urine proteins and pH. Kidney Int..

[33] Lieske JC, Swift H, Martin T, Patterson B, Toback FG (1994). Renal epithelial cells rapidly bind and internalize calcium oxalate monohydrate crystals. Proc Natl Acad Sci USA.

[34] Kanlaya R, Sintiprungrat K, Chaiyarit S, Thongboonkerd V (2013). Macropinocytosis is the major mechanism for endocytosis of calcium oxalate crystals into renal tubular cells. Cell Biochem. Biophys..

[35] Chaiyarit S, Singhto N, Thongboonkerd V (2016). Calcium oxalate monohydrate crystals internalized into renal tubular cells are degraded and dissolved by endolysosomes. Chem. Biol. Interact..

[36] Wiessner JH, Hasegawa AT, Hung LY, Mandel GS, Mandel NS (2001). Mechanisms of calcium oxalate crystal attachment to injured renal collecting duct cells. Kidney Int..

[37] Gan QZ, Sun XY, Bhadja P, Yao XQ, Ouyang JM (2016). Reinjury risk of nano-calcium oxalate monohydrate and calcium oxalate dihydrate crystals on injured renal epithelial cells: aggravation of crystal adhesion and aggregation. Int. J Nanomedicine..

[38] Selvaraju R, Thiruppathi G, Raja A (2012). FT-IR spectral studies on certain human urinary stones in the patients of rural area. Spectrochim. Acta A Mol. Biomol. Spectrosc..

[39] Selvaraju R, Raja A, Thiruppathi G (2015). FT-IR spectroscopic, thermal analysis of human urinary stones and their characterization. Spectrochim. Acta A Mol. Biomol. Spectrosc..

[40] Tomazic BB, Nancollas GH (1982). The dissolution of calcium oxalate kidney stones. A kinetic study. J. Urol..

[41] Tomazic BB, Nancollas GH (1979). A study of the phase transformation of calcium oxalate trihydrate-monohydrate. Invest Urol..

[42] Hedgepeth RC, Yang L, Resnick MI, Marengo SR (2001). Expression of proteins that inhibit calcium oxalate crystallization *in vitro* in the urine of normal and stone-forming individuals. Am. J Kidney Dis..

[43] O’Brien EP, Brooks BR, Thirumalai D (2012). Effects of pH on proteins: predictions for ensemble and single-molecule pulling experiments. J Am. Chem. Soc..

[44] Verkoelen CF, van der Boom BG, Houtsmuller AB, Schroder FH, Romijn JC (1998). Increased calcium oxalate monohydrate crystal binding to injured renal tubular epithelial cells in culture. Am. J Physiol.

[45] Gan QZ, Sun XY, Ouyang JM (2016). Adhesion and internalization differences of COM nanocrystals on Vero cells before and after cell damage. Mater. Sci Eng C Mater. Biol. Appl..

[46] Thongboonkerd V, Semangoen T, Chutipongtanate S (2006). Factors determining types and morphologies of calcium oxalate crystals: Molar concentrations, buffering, pH, stirring and temperature. Clin. Chim. Acta.

[47] Thongboonkerd V, Chutipongtanate S, Semangoen T, Malasit P (2008). Urinary trefoil factor 1 is a novel potent inhibitor of calcium oxalate crystal growth and aggregation. J Urol..

[48] Chaiyarit S, Mungdee S, Thongboonkerd V (2010). Non-radioactive labelling of calcium oxalate crystals for investigations of crystal-cell interaction and internalization. Anal. Methods..

[49] Chaiyarit S, Thongboonkerd V (2012). Changes in mitochondrial proteome of renal tubular cells induced by calcium oxalate monohydrate crystal adhesion and internalization are related to mitochondrial dysfunction. J Proteome. Res..

[50] Chutipongtanate S, Thongboonkerd V (2010). Systematic comparisons of artificial urine formulas for *in vitro* cellular study. Anal. Biochem..

[51] Fong-ngern K, Chiangjong W, Thongboonkerd V (2009). Peeling as a novel, simple, and effective method for isolation of apical membrane from intact polarized epithelial cells. Anal. Biochem..

[52] Fong-ngern K, Peerapen P, Sinchaikul S, Chen ST, Thongboonkerd V (2011). Large-scale identification of calcium oxalate monohydrate crystal-binding proteins on apical membrane of distal renal tubular epithelial cells. J Proteome. Res..

